# A misadventure of the correlation coefficient

**DOI:** 10.1016/j.tins.2022.09.009

**Published:** 2022-10-21

**Authors:** Dmitri A. Rusakov

**Affiliations:** 1 UCL Queen Square Institute of Neurology, University College London, London, UK

## Abstract

The correlation coefficient gauges linear association between two variables. However, interpreting its value depends on the question at hand. This article argues that relying on the correlation coefficient may be irrelevant for many neuroscience research tasks. When the experimental dataset is contextually suitable for binning-averaging, other indicators of statistical association could prove more suitable.

## Interpreting Pearson’s *R*

The commonly used Pearson’s correlation coefficient, Pearson’s *R*, evaluates linear association between two experimental variables, say *X* and *Y*. It has been widely accepted, at least across biological sciences, including neurobiology, that *R* > 0.7 indicates a strong linear relationship between *X* and *Y, R* < 0.3 weak, and the rest is moderate. This rule of thumb normally reflects the aim to predict the value of *Y* from measuring *X*, based on the *X*–*Y* relationship established for the population. The prediction reliability aspect of Pearson’s *R* follows from the fact that the *R*^*2*^ value, termed the coefficient of determination, stands for the proportion of the variation in *Y* that could be predicted from *X*. In essence, the correlation coefficient indicates how closely the data fit a linear pattern [1,2], which may explain practical usage of the foregoing traditional rule of thumb, particularly in clinical studies where prediction reliability is of importance.

However, in many documented cases these cut-off points appear arbitrary and inconsistent [2,3]. The correlation coefficient is of little importance unless it can be properly interpreted, which has been considered a difficult task for all scale values [1]. In fact, it has long been emphasised that the interpretation and usability of *R* depend on the specific question under study [2,4]. The use of *R* may be of particular relevance if the question falls into one of the following three categories: (i) How accurate is the prediction? (ii) What is the magnitude of error in a reliability task? (iii) What is the strength of the *X*–*Y* relationship? [4]. The present article will argue that, in many instances, neuroscience research tasks that involve the linear *X*–*Y* relationship fall outside this scope, so that referring to *R* appears irrelevant to the posed question.

## Cumulative effects arising due to a populational trend

In many experimental quests, predicting the *Y* value from measuring *X* for individual objects is not among the relevant, or even feasible, objectives. Such cases arise, for instance, when one or both variables show high inherent (biological) variability and/or when there has been a large measurement error. Multiple examples for this can be found in neurophysiology. In cellular neurophysiology, for example, a common objective is to understand whether cell behaviour is affected by some cumulative populational feature arising across thousands of synaptic connections. The *X*–*Y* datasets describing individual synapses normally display significant scatters. Such datasets may include, for instance, dendritic spine density against dendrite diameter [5]; local numbers of synaptic AMPA receptors against distance from the soma [6]; quantal content against a short-term plasticity parameter for glutamate release [7]; and so on. Here, the *X*–*Y* relationship among synapses varies too strongly to reliably predict *Y* from *X* for any individual synapse, which is duly reflected in low *R* values. It can be argued, however, that the focus of these studies is beyond the scope of questions relevant to *R*: here, given the unlimited numbers of realisations or events (synaptic inputs times synaptic discharges), relatively weak (but highly significant) positive correlations can have a clear cumulative effect, which is not duly captured via the analyses of *R*. For instance, a weak correlation between synaptic release probability and the distance from the synapse to the neuronal soma can result in a highly significant shift in synaptic signal summation leading to cell spiking [8].

In this context, the question is therefore not about the strength of the *X*–*Y* relationship *per se* but whether the *X*–*Y* relationship is statistically significant (i.e., whether it transpires over virtually unlimited sampling). Here, the magnitude of the physiological effect will depend not so much on the adherence of the *X*–*Y* scatter to the straight line, but rather on how steep the *X*–*Y* dependence is. A conventional estimator of this steepness is linear regression, where the regression coefficient (algebraic slope of the best-fit straight line) describes the expected magnitude of change in *Y* for a one-unit change in *X*. In such cases, referring to the *R* value as an indicator of the effect strength could be highly ambiguous, as illustrated later.

## Data binning-averaging increases *R* for the same dataset

**Figure 1:**
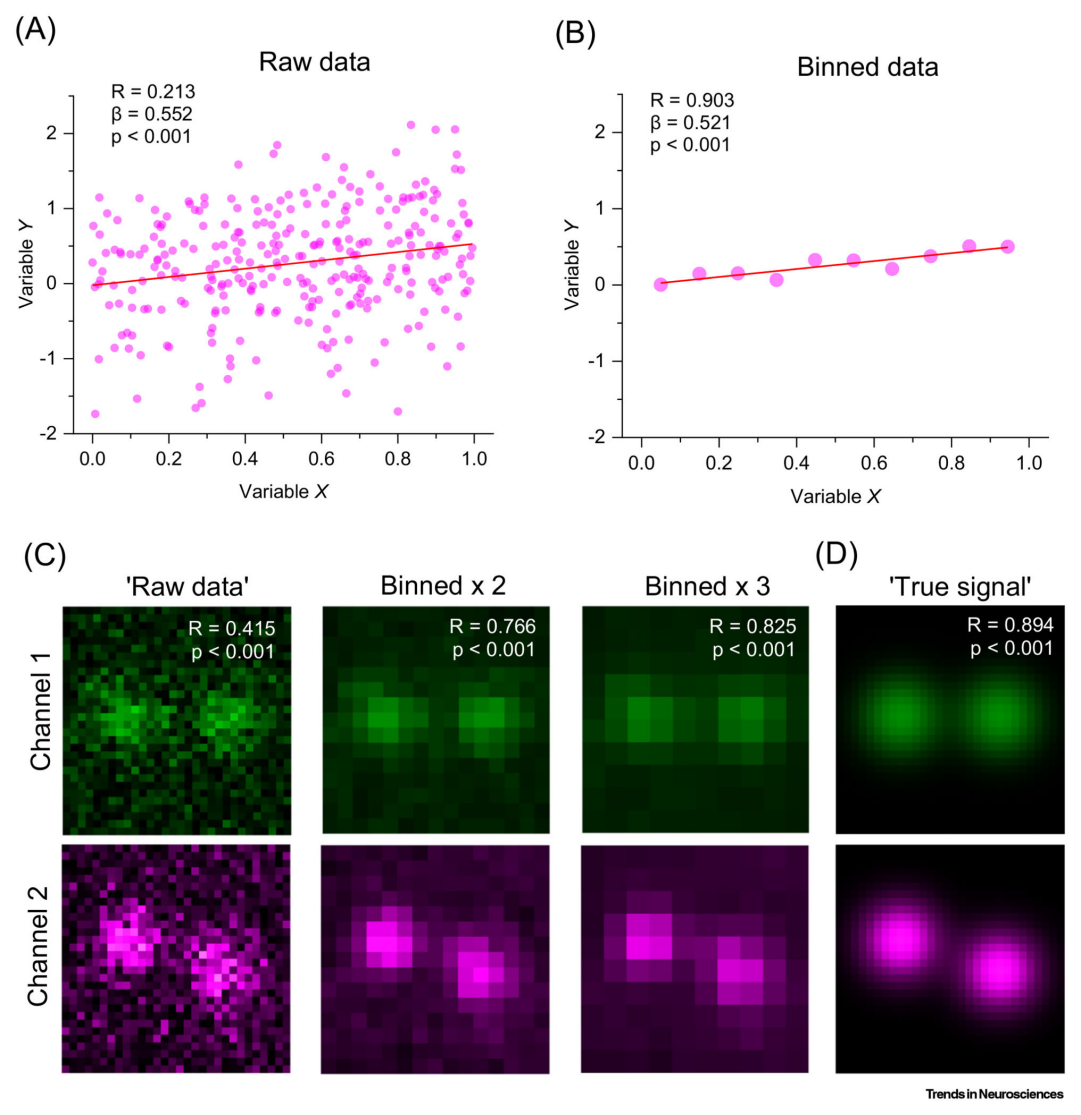
Binning raw data in the *X*–*Y* dataset elevates the correlation coefficient. Hypothetical datasets were computer generated to illustrate the effect of binning on Pearson’s correlation coefficient. (A) A Monte Carlo generated data set (*n* = 300 data points; OriginPro random number generator), with *X* values distributed uniformly randomly over [0; 1] interval and *Y* values following a Gaussian distribution centred at the slope line *Y* = β*X* (β = 0.5), with standard deviation σ = 0.7; unbroken line, best-fit linear regression; *R*, Pearson’s correlation coefficient; *P*, confidence level to reject the null-hypothesis ‘zero-slope of linear regression’. (B) The dataset as in (A), but binned with *Y* values averaged over 0.1 *X* intervals; *X* values shown at bin centres; other notations as in (A). (C) An example of Pearson’s *R* based image colocalisation analysis: computer generated brightness signals in a pair of image channels (green and magenta; Gaussian pixel noise added; ImageJ Fiji Process/Noise). The size of image pixels (binning) increases from left to right column as indicated. The Pearson’s *R* values for the interchannel pixel brightness colocalisation, and the statistical significance of the respective linear regression slope (H_0_-hypothesis rejection level p; ImageJ Fiji Colocalization/Coloc 2), are shown for the respective image pairs. (D) 'True signal': images as in (C) 'Raw data' panel but with all noise removed; notations as in (C).

The *X*–*Y* data scatter shown in Figure 1A is produced by generating a linear dependence between uniformly distributed *X* values over the 0–1 interval and *Y* values that follow the Gaussian distribution centred around the slope line (coefficient 0.5). This data scatter is illustrative of the experimental datasets mentioned earlier [5–7]: it reflects high data variability, low *R* value, yet highly significant regression (Figure 1A; β, linear regression coefficient). However, if we bin this dataset, by averaging *Y* values over the regular 0.1-long *X* intervals, we arrive at the reduced data scatter that shows an excellent linear relationship, with the strongly increased *R* but virtually unchanged β (Figure 1B). As an experimental example from work by the author and colleagues, a similar increase in *R* by data averaging can be shown in a dataset reporting release probability values versus distance from the synapse to the soma [8]. In the latter case, averaging (binning) synaptic features over a dendritic segment length reflects a realistic scenario when the bulk of synapses in that segment are activated [9]. Thus, essentially the same data sample could generate different *R* values, as long as the biological question under study permits data binning-averaging.

## Image binning and Pearson’s correlation

Pearson’s *R* has also been used to evaluate colocalisation or correlation between distributed signals recorded in two separate imaging channels, either static or dynamic (e.g., [10,11]). Digital images consist of pixels or voxels, the ‘elementary’ square or cubic areas over which the intensity of native signal sources (diffraction-limited light in case of light microscopy) is averaged. Thus, setting the pixel or voxel size by the imaging system is equivalent to the averaging-binning of raw data, as discussed earlier. It is no surprise, therefore, that Pearson’s *R* calculated to assess signal colocalisation between two images or image channels may depend on the pixel size [12]. The simulated example in Figure 1C illustrates a colocalisation test for a pair of image channels, each with two brightness spots representing the signal of interest; Gaussian noise has been added to reflect typical imaging conditions. Calculating Pearson’s *R* for signal colocalisation shows that its value increases strongly with image binning (increasing pixel size; Figure 1C). This increase does not appear spurious because the ‘true signal’ (noise removed) corresponds to the highest *R* (Figure 1D). At the same time, the linear correlation slope remains significantly above zero throughout (*P* < 0.001), suggesting that colocalisation is statistically significant. However, quantifying the strength of signal colocalisation in such cases is a complex, context-dependent task, which is outside the scope of this article. Here we simply argue that relying on Pearson’s *R* may not be the most robust approach for establishing signal correlation or colocalisation for multiplexed imaging.

## Concluding remarks

This article outlined some basic considerations around the use of Pearson’s *R* and argues against overinterpreting Pearson’s *R* as an unequivocal indicator of the ‘strength’ or ‘weakness’ of the effect arising from the linear dependence of two variables. In some experimental designs where the task in question permits data binning or averaging, as often encountered in neuroscience, a regression analysis should provide more reliable insights. In this context, it might be particularly ambiguous to rely on *R* in the case of experimental metrics with inherent binning-averaging, such as variable pixel/voxel values used in cellular or brain imaging.
